# ConoMode, a database for conopeptide binding modes

**DOI:** 10.1093/database/baaa058

**Published:** 2020-08-04

**Authors:** Xiao Li, Hao Liu, Chunxiao Gao, Yangyang Li, Dongning Jia, Yanbo Yang, Jinbo Yang, Zhiqiang Wei, Tao Jiang, Rilei Yu

**Affiliations:** 1 Key Laboratory of Marine Drugs, Chinese Ministry of Education, School of Medicine and Pharmacy, Ocean University of China, Qingdao 266003, China; 2 Department of Computer Science and Technology, Ocean University of China, Qingdao 266100, China; 3 Qingdao National Laboratory for Marine Science and Technology, Qingdao 266003, China

## Abstract

ConoMode is a database for complex three-dimensional (3D) structures of conopeptides binding with their target proteins. Conopeptides, a large family of peptides from the venom of marine snails of the *Conus* genus, have exceptionally diverse sequences, and their high specificity to block ion channels makes them crucial as drug leads and tools for physiological studies. ConoMode is a specialized archive for the collection of 3D coordinate data for the conopeptides and their binding target proteins from published literature and the Protein Data Bank. These 3D structures can be determined using experimental methods such as X-ray crystallography and electron microscopy and computational methods including docking, homology modeling and molecular dynamics simulations. The binding modes for the conopeptides determined using computational modeling must be validated based on experimental data. The 3D coordinate data from ConoMode can be searched, visualized, downloaded and uploaded. Currently, ConoMode manages 19 conopeptide sequences (from 10 *Conus* species), 15 protein sequences and 37 3D structures. ConoMode utilizes a modern technical framework to provide a good user experience on mobile devices with touch interaction features. Furthermore, the database is fully optimized for unstructured data and flexible data models.

**Database URL**: http://conomode.qnlm.ac/conomode/conomode/index

## Introduction

Conotoxins are cocktail-like mixtures derived from the venom of marine predatory cone snails, which contains more than 100 types of bioactive peptides (1, 2). It is estimated that ~1 000 000 conotoxins exist among different *Conus* species (3), and they can be categorized according to the cysteine framework, gene superfamily and pharmacological family (2, 4). The majority of conotoxins range in sequence length from 10 to 40 amino acids (4), and their highly conserved disulfide linkages, backbones and diverse chemical structures enable their excellent potency and specificity for various targets, including nicotinic acetylcholine receptors (nAChRs), ion channels (sodium, calcium, potassium), G-protein coupled receptors and enzymes (5, 6). Conotoxins have remarkable potential for therapeutics, making them valuable as pharmacological tools and peptide drug leads. ω-Conotoxin MVIIA, also known as ziconotide, was approved by the U.S. Food and Drug Administration as an effective analgesic drug to treat chronic nociceptive and neuropathic pain of malignant and nonmalignant origin (7). The identification of interactions between conotoxins and their targets will substantially facilitate the development of new drugs.

**Figure 1.**
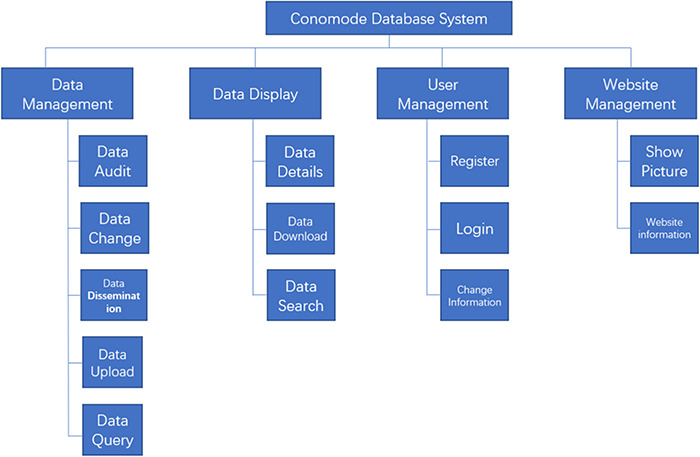
Structure and function of the ConoMode webserver.

**Figure 2.**
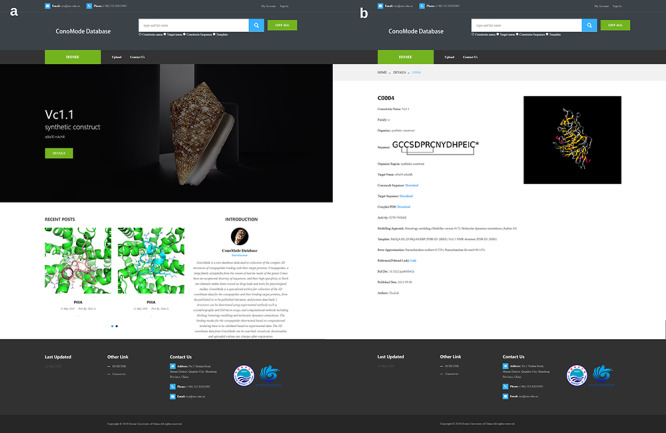
Screenshot of ConoMode website. (**a**) Interface of the ConoMode database. (**b**) Detailed page of Vc1.1-α9α9.

α-Conotoxins, a major conotoxin family, are typical antagonists of nAChRs (6, 8). nAChRs function as ligand-gated ion channels, which are generally responsible for synaptic transmission in the nervous system or other nonneural cells in different physiological events (6, 8, 9). nAChRs can be inhibited by conotoxins, producing analgesic effects in inflammatory pain and hyperalgesia neuropathic pain models (9). Crystal structures of the acetylcholine binding protein (AChBP), a homolog of the nAChR extracellular domain, have been used as surrogate or homology modeling templates to study the interactions between conotoxins and nAChRs (10–17).

Another large pharmacological class of conotoxins are μ-conotoxins, which exhibit high potency and specificity on voltage-gated sodium channels (Navs) (18–22). Navs initiate action potentials in neurons and other excitable cells, and their dysfunction causes inherited epilepsy, chronic pain and other diseases of hyperexcitability (18). Since sodium channels play a key role in generating and propagating action potential, they represent a class of appealing targets for drug development in the treatment of neuropathic pain (10, 18). Recently, crystal structures of prokaryotic Navs, such as those from *Arcobacter butzleri* (NavAb) and *Magnetococcus marinus* (NavMs) (20, 22), have also been used extensively as templates for the homology modeling of eukaryotic Navs interacting with μ-conotoxins (21, 22).

Owing to their potential druggability and lower cellular toxicity compared to that of small-molecule drugs, conotoxins are receiving increasing attention based on their development as drug leads (1, 9). Different chemical modification strategies have been used to enhance the potency, specificity and stability of conotoxins (1). However, the structure-activity relationship of conotoxins interacting with ion channels is poorly understood because of the lack of high-resolution complex structures between conotoxins and their targets. Determination of ion channel crystal structures remains a challenge, whereas crystal structures of their homologs, such as AChBP and NavAb or NavMs are relatively easy to determine.

Recent advancements in cryo-electron microscopy (cryo-EM) technology have made it possible to resolve the relatively high-resolution structure of ion channels, such as nAChR, eukaryotic Nav and voltage-gated calcium channels (23–27). The appearances of these structures significantly increased the accuracy of computationally modeling the interactions between these ion channels and their targeting conotoxins (29–32). To date, only a few conotoxin-bound ion channel structures are present in the Protein Data Bank (PDB) (23, 28). Thus, computational modeling of the interactions between conotoxins and their targeting receptors will continue to be the most efficient approach for understanding the structure-activity relationship of conotoxins at the molecular level (29, 30).

Accurate determination of the binding modes of conotoxins to their targeting ion channels or receptors is essential for understanding their structure-activity relationship as well as for efficiently enhancing their activity, specificity or stability (2). Recently, more than 50 computational models between conotoxins and their targeting ion channels were published, among which more than 20 were published by Yu *et al*. (33–40). Most of these models were supported by their ability to explain results from mutagenesis studies. These models could be used to interpret experimental results or even as guidance for structure optimization of these conotoxins. However, these complex structures are not accepted by the PDB database, in which only structures determined based on crystallography, cryo-EM or nuclear magnetic resonance (NMR) are accepted for deposition (23, 28). Thus, a database for specifically managing giving access to these published conotoxin/ion channel structures would be helpful to the community working on peptide toxins. ConoMode is a database that specifically collects the binding complexes between conotoxins and their targets generated from computational modeling or determined based on experimental approaches, and a specialized administrator is responsible for regular updates and management at the Pilot National Laboratory of Marine Science and Technology (Qingdao). Users can freely download existing complexes or upload new complexes, which will significantly contribute to research on the conotoxin mechanism of action.

## Materials and Methods

### Data collections

Currently, a total of 37 modes of binding between conotoxins and nAChRs or ion channels are exhibited on the ConoMode website. Among them, 28 were derived from our previously published studies in recent years (33–40). These 28 structures were determined using computational modeling strategies, including homology modeling, docking and molecular dynamics simulations and the other 9 were derived from crystal complex structures obtained from the PDB (IDs: 4EZ1, 5CO5, 2C9T, 5XGL, 5JME, 2BR8, 2UZ6, 2BYP, 5T90, 6J8E) (10–17, 23, 28).

### Database architecture and web interface

To support the search for a complex structure and for such an uncertain data model, the technical framework of the ConoMode webserver uses a typical SSM (Spring + SpringMVC + MyBatis) architecture (Figure 1). The webserver utilizes schema-free technology to store data, and the model of the data entity can be flexibly changed according to requirements. Furthermore, ConoMode is optimized for unstructured data, especially conotoxin structures, and all file information content is dynamically generated by the database in the system.

## Raw data uploading process

The ConoMode database will be enriched by collecting the published complex structures of conotoxins/ion channels or receptors determined based on experimental approaches or calculated via computational modeling.

Registered users can submit details of a complex structure between conotoxins and their targets. The uploading process requires three fundamental fields: the complex in PDB format, conotoxin sequence and target sequence. Users can provide source references and comments as extra fields. All successfully uploaded fields will be reviewed based on published experimental data. The evaluation consists of the data format and quality of the model. All accepted data will be deposited in the ConoMode database and will be displayed on the website.

In ConoMode, each conotoxin complex is assigned a new ID according to the order in which it is uploaded. For instance, ‘C0001’ represents the first complex uploaded on the website, and “C” is the initial of ‘conotoxin’.

## Search and visualization

Currently, ConoMode supports the conotoxin name, target name and conotoxin sequence as search keywords (Figure 2). The results with brief information and link details are presented as a table. On the detailed page of each conotoxin, we provide download links for the complex, conotoxin sequence and target sequence. Information is divided into three parts on the webpage. The first part contains basic information on conotoxins, such as its name, sequence, organism species and original region (3, 4). The second part consists of modeling approach, modeling templates and error approximation, the digital object identifier and the abstract of the references that the complex structure was determined was showed in the third part.

Target names and IC_50_ values constitute the major content of the third part. However, not every complex has exact IC_50_ values, especially those with binding modes obtained from computational modeling; therefore, this information is not always available on the website.

Considering that it is challenging to obtain the crystal structure of conotoxin complexes by experimental methods, ConoMode provides a special library for archiving conotoxin complexes determined by computational modeling. Users can not only use the models in ConoMode but also can share their published models with other researchers.

## Discussion

ConoMode is an unprecedented database that systematically collects complex structures of conotoxins and targets. Considering that it is challenging to obtain the crystal structure of a complex by experimental methods, ConoMode provides a special library of binding modes determined by homology modeling and molecular dynamics simulation for users to search, and users can also share their published models. Furthermore, interactions between conotoxins and their targets can be well predicted through these binding models, which makes ConoMode a powerful tool to promote research in this field.
